# The Pattern of Complaints about Australian Wind Farms Does Not Match the Establishment and Distribution of Turbines: Support for the Psychogenic, ‘Communicated Disease’ Hypothesis

**DOI:** 10.1371/journal.pone.0076584

**Published:** 2013-10-16

**Authors:** Simon Chapman, Alexis St. George, Karen Waller, Vince Cakic

**Affiliations:** Sydney School of Public Health, University of Sydney, New South Wales, Australia; University of Florida, United States of America

## Abstract

**Background and Objectives:**

With often florid allegations about health problems arising from wind turbine exposure now widespread, nocebo effects potentially confound any future investigation of turbine health impact. Historical audits of health complaints are therefore important. We test 4 hypotheses relevant to psychogenic explanations of the variable timing and distribution of health and noise complaints about wind farms in Australia.

**Setting:**

All Australian wind farms (51 with 1634 turbines) operating 1993–2012.

**Methods:**

Records of complaints about noise or health from residents living near 51 Australian wind farms were obtained from all wind farm companies, and corroborated with complaints in submissions to 3 government public enquiries and news media records and court affidavits. These are expressed as proportions of estimated populations residing within 5 km of wind farms.

**Results:**

There are large historical and geographical variations in wind farm complaints. 33/51 (64.7%) of Australian wind farms including 18/34 (52.9%) with turbine size >1 MW have never been subject to noise or health complaints. These 33 farms have an estimated 21,633 residents within 5 km and have operated complaint-free for a cumulative 267 years. Western Australia and Tasmania have seen no complaints. 129 individuals across Australia (1 in 254 residents) appear to have ever complained, with 94 (73%) being residents near 6 wind farms targeted by anti wind farm groups. The large majority 116/129(90%) of complainants made their first complaint after 2009 when anti wind farm groups began to add health concerns to their wider opposition. In the preceding years, health or noise complaints were rare despite large and small-turbine wind farms having operated for many years.

**Conclusions:**

The reported historical and geographical variations in complaints are consistent with psychogenic hypotheses that expressed health problems are “communicated diseases” with nocebo effects likely to play an important role in the aetiology of complaints.

## Introduction

The attribution of symptoms and disease to wind turbine exposure is a contentious “modern health worry” [1] which has seen increasing attention from governments, their regulatory agencies and courts after organised opposition to wind farms, predominantly in Anglophone nations. Two broad hypotheses have been advanced about those reporting symptoms they attribute to exposure to wind turbines.

1. both audible noise and sub-audible infrasound generated by wind turbines can be directly harmful to the health of those exposed.
2. psychogenic factors – including nocebo responses to the circulation of negative information about their putative harms – are likely to be relevant to understanding why of those exposed, only small proportions claim to be adversely affected.

The evidence for a physical basis for these symptoms remains largely anecdotal. There has been a profusion of claims mostly by wind farm opponents about harms to exposed humans and animals (currently numbering 223 different diseases and symptoms) [2]. Despite this, 18 reviews of the research literature on wind turbines and health published since 2003 [3]–[20] have all reached the broad conclusion that the evidence for wind turbines being directly harmful to health is very poor. These suggest that only small minorities of exposed people claim to be annoyed by wind turbines – typically less than 10% [14]. They conclude that the relationship between wind turbines and human responses is “influenced by numerous variables, the majority of which are non-physical” [14].

Variables associated with wind turbine annoyance include pre-existing negative attitudes to wind farms [14], including their impact on landscape aesthetics [21], having a “negative personality” [22], subjective sensitivity to noise [14], and being able to see wind turbines [5], [23]. Similarly, deriving income from turbines [24] or enjoying reduced power bills can have an apparent “protective effect” against annoyance and health symptoms [18]. Such factors, which are similar to characteristics of other psychogenic illnesses (“New Environmental Illnesses” [25] and “Modern Health Worries” [26]) were found to be more predictive of symptoms than objective measures of actual exposure to sound or infrasound [14].

A large literature on nocebo effects exists about reported pain [27], but these effects have also been documented for other imperceptible agents such as electro-magnetic and radio frequency radiation [28]–[30]. Perceived proximity to mobile telephone base stations and powerlines, lower perceived control and increased avoidance (coping) behaviour were associated with non-specific physical symptoms in a study which found no association between reported symptoms and distance to these sources of electromagnetic radiation [31].

The psychogenic theory about wind turbine “illness” is supported by a recent New Zealand study [32], in which healthy volunteers exposed to both sham and true recorded infrasound who had been previously given information about possible adverse physiological effects of infrasound exposure reported symptoms aligned with that information. The adverse effects information provided to subjects was sourced from anti wind farm internet sites which the authors concluded indicated “the potential for symptom expectations to be created outside of the laboratory, in real world settings.”

A psychogenic contagion model may be applicable to this phenomenon. Mass Psychogenic Illness (MPI) is described [33]–[35] as a constellation of somatic symptoms, suggestive of an environmental cause or trigger (but with symptoms without typical features of the contaminant, varying between individuals, and not related to proximity or strength of exposure) which occurs between two or more people who share beliefs related to those symptoms and experience epidemic spread of symptoms between socially connected individuals. The rapid development of fear and anxiety is key to the transmission of disease by disruption of behaviour and activities of those involved. Transmission or contagion is increased by the general excitement related to the phenomenon, including media reports, researcher interest, and labeling with a specific clinical diagnostic term.

Boss’ review of factors promoting mass hysteria noted that “media reports are used as cues by potential cases for appropriate illness behavior responses and can initially alarm those at risk …Too often, it is the media-created event to which people respond rather than the objective situation itself … Development of new approaches in mass communication, most recently the Internet, increase the ability to enhance outbreaks through communication.” [33].

While modern wind farms have operated since the early 1980s [36], the earliest claims alleging that wind turbines might cause health problems in those exposed appear to date from 2003 (see below); this increased rapidly after 2008, following publicity given to a self-published book, “Wind Turbine Syndrome” [37], by US physician Nina Pierpont, whose partner edits a virulent anti wind farm website [38]. Google Trends data of web-based searches for “Wind turbine noise”, “Wind Turbine Syndrome” and “wind turbine health” show that “noise” began to appear from 2007 and that “syndrome” and “health” began to track together from 2008, suggesting the book generated this sudden interest in the phenomenon, rather than riding a wave of interest. Furthermore, a 2007–11 Ontario study of newspaper coverage of wind farms showed that 94% of articles featured “dread” themes [39].

“Labeling” of an illness is one of the key features associated with spread of mass psychogenic illness, along with community and media interest [33]. There have been three attempts to popularise portentous quasi-scientific names for health problems said to be caused by wind turbines: Wind Turbine Syndrome, Vibro Acoustic Disease [40] and Visceral Vibratory Vestibular Disturbance [41], although none of these have gained scientific acceptance as diagnostic terms. As described earlier, many features of MPI apply to Wind Turbine Syndrome. Furthermore, the most reported symptoms in over one third of all MPIs of nausea/vomiting, headache, and dizziness [33], are also frequently featured as common symptom complaints arising with wind turbines, suggesting these symptoms may be plausibly explained as psychogenic.

Wind farm opponent groups have been very active in the last five years in three Australian states (Victoria, NSW and South Australia) publicising the alleged health impacts of turbines. This has created insurmountable problems for researching the psychogenic and nocebo hypotheses using either cross-sectional or prospective research designs because it is unlikely that any communities near wind farms now exist which have not been exposed to extensive negative information. For this reason, audits of the history of complaints are essential because they allow consideration of whether health and noise complaints arose during years prior to the “contagion” of communities with fearful messages about turbines.

To date, there has been no study of the history and distribution of noise and health complaints about wind turbines in Australia. The two theories (the “direct effects” and the “psychogenic”), would predict differing patterns of spatial and temporal spread of disease. We sought to test 4 hypotheses relevant to the psychogenic argument.

1. Many wind farms of comparable power would have no history of health or noise complaints from nearby residents (suggesting that exogenous factors to the turbines may explain the presence or absence of complaints).
2. Wind farms which have been subject to complaints would have only a small number of such complaining residents among those living near the farms (suggesting that individual or social factors may be required to explain different “susceptibility”).
3. Few wind farms would have any history of complaints consistent with claims that turbines cause acute health problems (suggesting that explanations beyond turbines themselves are needed to explain why acute problems are reported).
4. Most health and noise complaints would date from after the advent of anti wind farm groups beginning to foment concerns about health (from around 2009) and that wind farms subject to organised opposition would be more likely to have histories of complaint than those not exposed to such opposition (suggesting that health concerns may reflect “communicated” anxieties).

Table 1 sets out both the predictions of the “direct effects” model of causation, and the observed findings of our historical review of the distribution and timing of complaints, which are more consistent with a psychogenic model.

**Table 1:** Prediction of “direct effects” model versus observations explained by psychogenic model.

Key hypotheses re distributionof complainants	Characteristic	Predictions of DirectEffects Model	Observations withPsychogenic Model
Spatial (geographic)	Distribution of wind farmswith complaints	All wind farms (especially those with>1 MB turbines) should havecomplainants	Inconsistent distribution associated withpresence or absence of anti windfarm activity
	Proportion of complainantsresiding around wind farms	Only in those “susceptible” but shouldbe similar across all wind farms	Generally very low, but higher at windfarms targeted by anti windfarm groups
Temporal	Timing and latency offirst complaints	Turbine exposure followed by bothacute (immediate) and chronichealth effects	Absence of or long delays in reportingacute effects common

## Methods

Information on the commencement of turbine operation, the number of turbines operating, average turbine size and the megawatt (MW) capacity of each wind farm was located from public sources such as wind farm websites.

Wind farm operators have clear risk management interest in any reactions of nearby residents to the farms they operate. In the planning, construction and power generation phases of wind farm operation they monitor local community support and complaints submitted to them, in news media and via any complaint notifications from local government. In Victoria, companies are required by law to register all complaints with the state government. In September 2012 all wind farm owners in Australia were asked to provide information on:

- the actual or estimated number of residents within a 5 km radius of each wind farm they operated. Google Maps and census data were also used to obtain this data (see below).
- whether the company had received or was aware of any health and/or noise complaints, including sleeping problems, that were being attributed to the operation of their wind farms.
- the number of individuals (“complainants”) who had made such complaints (direct complaints to the companies, those voiced in local media, to local government or state or national enquiries).
- the date at which the first complaint occurred.
- whether there had been any anti wind farm activity in the local area such as public meetings addressed by opponents, demonstrations or advertising in local media.

Any documentation of complaints such as internet links or news clips about public was requested. Companies were explicitly asked to de-identify any private complaints which could identify those complaining, unless these complaints had been made public by the complainants.

It is possible that wind companies may nonetheless be unaware of some health and noise complaints about their operations or that they might downplay the extent of complaints and provide underestimates of such complaints. To corroborate the information on the number of complainants provided by the companies, we therefore reviewed all 1,594 submissions made to three government enquiries on wind farms: the 2011–2012 Senate enquiry into the Social and Economic Impact of Rural Wind Farms (1,818 submissions) [42]; the 2012 NSW Government’s Draft NSW Planning Guidelines for Wind Farms (359 submissions) [43]; and the Renewable Energy (Electricity) Amendment (Excessive Noise from Wind Farms) Bill 2012 (217 submissions) [44]. We searched all submissions for any mentions by residents living in the vicinity of operating wind farms (as opposed to those being planned) of their health or sleep being adversely affected or that they were annoyed by the sound of the turbines.

We also searched daily media monitoring records supplied to the Clean Energy Council by a commercial monitoring company from August 2011 (when the monitoring contract began) until January 2013. This monitoring covered print news items, commentary and letters published in Australian national, state and regional newspapers mentioning any wind farm, as well as television and radio summaries about all mentions of wind farms. It was important to use this source of monitoring rather than use on-line databases like Factiva, as the latter do not cover all small rural news media which is where much coverage of debate about rural wind farms was likely to be found.

Finally, a pre-print of this paper was published on the University of Sydney’s e-scholarship repository on March 15 2013. In the next six months the paper was opened over 10,800 times, making it the most opened document among 7761 in that repository across these 4 months. This generated considerable correspondence, and in one case (Hallett 2), information was provided about extra complainants who had complained via a legal case. These were then included.

In reviewing the submissions and media monitoring, only complaints from those claiming to be personally affected by the operation of an existing wind farm in Australia were noted. Expressed concerns about possible future adverse effects or that wind turbines *could* be harmful were not classified as evidence of personal experience of harm or annoyance. There were many of these. Third party statements, such as comments about unnamed neighbours with problems, were not accepted as evidence of harm.

Where the numbers of complainants determined from this corroborative public source searching exceeded the numbers provided to us by the wind companies, we chose the larger number. Where the numbers determined from public sources were less, we used the larger number provided by the companies. Our estimate of the number of complainants thus errs on the least conservative side. Nearly all those who publicly complained did not seek anonymity, being named in media reports or not electing to have their parliamentary submissions de-identified. However, we have chosen not to list their names in this report.

The companies provided estimates of the number of residents currently living within 5 km of each wind farm. Some companies provided estimates of the number of individuals, while others provided data on the number of houses. In Table 2, we have multiplied cells showing the number of *houses* by 2.6, this being the average number of residents per household in Australia today, to give a total estimate of surrounding residents.

**Table 2:** Complainant numbers at 51 Australian wind farms, 1993–2013.

Wind farm name (state)*owner*	Installed Capacity(MW)+(number ofturbines)+averageturbine size MW	Date commencedoperation & totalyears (to Dec 2012)	Approx. populationwithin 5 km	Health or noisecomplainants (Y/N)& number (personsunless specified)	Date of firstcomplaint (monthssince opened)	Local or visitingopposition groupactivity?
**A: Farms with total >**1**0** **MW capacity**						
Albany/Grasmere (WA)*Verve*	35.4 (18)1.96	Oct 2001(11y 2m)	200	N	–	N
Bungendore/Capital/Woodlawn (NSW) *Infigen*	189 (90)2.1	Nov 2009(3y 1m)	76 houses198	Y:10	Dec 2009(1 m)	Y
Canunda (SA)*International Power*	46 (23)2.0	Mar 2005(7y 10m)	20 houses52	N	–	N
Cape Bridgewater (Vic)*Pacific Hydro*	58 (29)2.0	Nov 2008(4y 1m)	68 houses177	Y:6	2 Feb 20110(16m)	Y
Cape Nelson South (Vic)*Pacific Hydro*	44 (22) 2.0	Jun 2009(3y 6m)	170 houses425	Y:2	10 Feb 2010(8m)	Y
Cathedral Rocks (SA)*TRUenergy, Acciona &* *EHN*	66 (33)2.0	Sep 2005(7 y 3 m)	0	N	–	N
Challicum Hills (Vic)*Pacific Hydro*	52.5 (35)1.5	Aug 2003(9 y 4 m)	55 houses143	N	–	N
Clements Gap (SA)*Pacific Hydro*	56.7 (27)2.1	Feb 2010(2 y 10 m)	41	Y:3	On-going from earlier	Y
Codrington (Vic)*Pacific Hydro*	18.2 (14)1.3	Jun 2001(11 y 6 m)	50	N		N
Collgar/Merriden (WA)*Collgar*	206 (111)1.85	May 2011(1 y 7 m)	15	N	–	N
Cullerin Range (NSW)*Origin*	30 (15)2.0	Jul 2009(3 y 5 m)	50	N	–	N
Emu Downs (WA)*APA*	80 (48)1.66	Oct 2006(6 y 2 m)	50	N	–	N
Gunning/Walwa (NSW)*Acciona*	46.5 (31)1.5	May 2011(1 yr 7 m)	25 houses65	Y:1	Jan 2012(8 m)	N
Hallett 1/Brown Hill (SA)*AGL*	95 (45)2.11	Sep 2008(4 y 3 m)	120	N		Y
Hallett 2/Hallett Hill (SA)*AGL*	71.4 (34)2.1	Mar 2010(2 y 9 m)	120	Y:13*	On-going from earlier	Y
Hallett 4/North Brown Hill (SA)*AGL*	132 (63)2.1	May 2011(1 y 7 m)	200	Y:1	On-going from earlier	Y
Hallett 5/Bluff Range (SA)*AGL*	53 (25)2.1	Mar 2012(9 m)	140	Y:1	Apr 2012(1 m)	Y
Lake Bonney (SA)*Infigen*	278.5 (112)2.8	Mar 2005(7 y 9 m)	255	Y:2	June 2012(7 y 3 m)	N
MacArthur (Vic) *AGL/* *Meridian*	420 (140)3.0	Sep 2012(3 m)	15	Y:8 houses = 21	2 days after 2/140 turbines commenced operation	Y
Mortons Lane (Vic) *CGN* *Wind Energy Ltd*	19.5 (13)1.5	Dec 2012	14 houses36	N	–	N
Mt Millar (SA)*Meridian*	70 (35)2.0	Feb 2006(6 y 10 m)	10 houses26	N	–	N
Oaklands Hill (Vic)*AGL*	67.2 (32)2.1	Feb 2012(10 m)	250	Y:6	On-going from earlier	Y
Snowtown (SA)*Trust Power*	100.8 (47)2.14	Nov 2008(4 y 1 m)	4 houses10	N	–	N
Starfish Hill (SA)*Ratch*	34.5 (23)1.5	Sep 2003(9 y 3 m)	200	N	–	N
Toora (Vic)*Ratch*	21 (12)1.75	Jul 2002(10 y 5 m)	674	Y:2	Early (precise date not known)	Y
Walkaway (Alinta) (WA)*Infigen*	89.1 (54)1.65	Apr 2006(6 y 8 m)	3 houses8	N	–	N
Waterloo (SA)*TRUenergy*	111 (37)3.0	Dec 201(2 y)	75 houses195	Y:11	Feb 2011(2 m)	Y
Wattle Point (SA)*AGL Hydro*	91 (55)1.65	Nov 2005(7 y 1 m)	560	N	–	N
aubra (Vic)*Acciona*	192 (128)1.5	Mar 2009(3 y 10 m)	283 houses736	Y:29	13 Mar 2009 (immediate)	Y
Windy Hill (Qld)*Ratch*	12 (20)0.6	Feb 2000(12 y 10 m)	200	Y:1	Early (precise date not known)	N
Wonthaggi (Vic)*Transfield*	12 (6)2.0	Dec 2005(7 y)	6900	Y:∼10	Feb 2006(2 m)	Y
Woolnorth:Bluff Point (Tas) *Roaring 40* *s* *& Hydro Tas*.	65 (37)1.76	Aug 2002(10 y 4 m)	NI	N	–	N
Woolnorth:Studland Bay (Tas) *Roaring 40* *s* *& Hydro Tas*.	75 (25)3.0	May 2007(5 yr 7 m)	NI	N	–	N
34.Yambuk (Vic) *Pacific* *Hydro*	192 (128)1.5	Jan 2007(5 y 11 m)	88	N	–	N
Sub-total: 34 farms	3130.3 MW (1567 turbines)		12334	16 farms with 119 complainants		14
**B: Farms with <10** **MW capacity**						
Blayney (NSW)*Eraring Energy*	9.9 (15)0.66	Oct 2000(12 y 2 m)	37	N	–	N
Bremer Bay (WA)*Verve*	0.6 (1)0.6	Jun 2005(7 y 6 m)	250	N	–	N
Coober Pedy (SA)*Energy Generation*	0.15 (1)0.15	1999(13 y)	3500	N	–	N
Coral Bay (WA)*Verve*	0.825 (3)0.275	Oct 2006(6 y 2 m)	200	N	–	N
Crookwell (NSW)*Union Fenosa/Eraring*	4.8 (8)0.6	Jul 1998(14 y 5 m)	200	Y:4	Jan 2012(13 y 6 m)	Y
Denham (WA)*Verve*	1.6 (4)0.4	Jun 1998(14 y 6 m)	600	N	–	N
Esperance, 9 Mile Beach (WA) *Verve*	3.6 (6)0.6	2003(8 y)	50	N	–	N
Esperance, 10 Mile Lagoon (WA) *Verve*	2.025 (9)0.225	1993(19 y)	50	N	–	N
Hampton Park (NSW) *Wind Corp*	1.32 (2)0.66	Sep 2001(11 y 3 m)	150	N	–	N
Huxley Hill, King Island (Tas) *Hydro Tas*	2.458 (5)0.49	Feb 1998(14 y 1 m)	10 houses(26)	N	–	N
Hopetoun (WA)*Verve*	1.2 (2)0.6	Mar 2004(8 y 9 m)	600	N	–	N
Kalbarri (WA)*Verve*	1.6 (2)0.8	Jul 2008(4 y 5 m)	10	N	–	N
Kooragang, Newcastle (NSW) Energy Australia	0.6 (1)0.6	1997(15 y)	3–4 km from Mayfield9000	N	–	N
Leonards Hill (Vic) *Community owned*	4.1 (2)2.05	Jun 2011(1 y 6 m)	232	Y:6	On-going from earlier	Y
Mt Barker (WA)*Mt Barker Power*	2.4 (3)0.8	Mar 2011(1 y 9 m)	2000	N	–	N
Rottnest Island (WA) *Rottnest Island*	0.6 (1)0.6	Sep 2006(6 y 3 m)	150	N	–	N
Thursday Island (Qld) *Egon Energy*	0.225 (2)0.113	Aug 1997(15 y 5 m)	2500	N	–	N
Sub-total:17 farms	38 MW67 turbines		20405	2 farms with 10 complainants		2
Total:51 farms	3168.3 MW1634 turbines		32739	18 farms with 129 complainants		16

NI =  no information.

* 13 residents submitted affidavits in a court case but only 2 complained to the company (*AGL*), and none to the local Council or Environmental Protection Agency.

Average residents per house in 2011∶2.6 http://www.censusdata.abs.gov.au/census_services/getproduct/census/2011/quickstat/0.

## Results

Table 2 shows the history and distribution of complaints from all 51 Australian wind farms. Complaints came either from individuals or from households with several occupants each or collectively complaining. Some wind companies initially reported the number of complainants as *households*, while others reported individual complainant numbers. In these cases we sought clarification from companies about whether complaints came from single individuals, couples or more than two members of a family so as to report total the estimated total number of individual complainants.

### Hypothesis 1: Many Wind Farms would have no History of Complaints

Of all 51 wind farms, 33 (64.7%) had never been subject to health or noise complaints, with 18 (35.3%) receiving at least one complaint since operations commenced. The 33 farms with no histories of complaints, and which today have an estimated 21,633 residents living within 5 km of their turbines, have operated for a cumulative total of 267 years.

Of the 18 wind farms which had received complaints, 16 were larger wind farms (≥10 MW capacity). In summary, 18/34 (52.9%) of larger wind farms, and 15/17 (88.2%) of small farms have never experienced complaints. Wind farm opponents sometimes argue that it is mainly very large, “industrial” wind turbines which generate sufficient audible noise and infrasound to cause annoyance and health problems. If 1 MW is taken to define a “large” turbine, 18/34 (52.9%) of farms using large turbines had never attracted complaints while 15/17 (88%) of farms using smaller turbines had no histories of complaints. Both the total energy generating capacity of farms and whether the turbines used were over 1 MW were thus significant predictors of residents having ever complained, with small total capacity farms being far less likely to have complainants (88% vs 53%; χ^2^ = 6.18, 1 df, p = 0.013).

**Figure 1:**
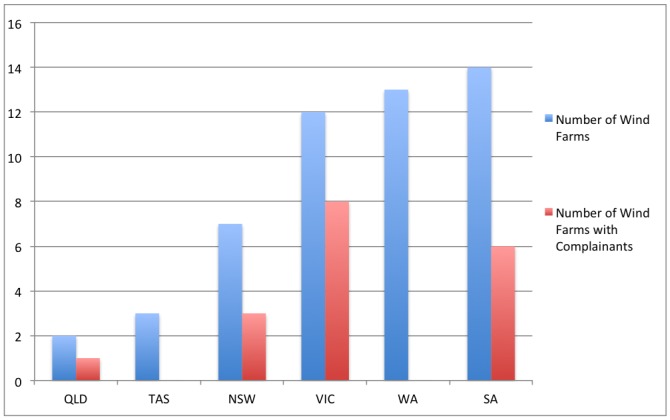
Farms with wind turbine complainants by state, Australia 1993–2012.

The distribution of farms which have ever received complaints is highly variable across Australia. Figure 1 shows no consistency between the percentages of farms receiving complaints in different states, whether they have many or few wind farms. Western Australia has 13 wind farms (3 with large turbines), including some of the longest running in Australia (Esperance 10 Mile Lagoon 1993, Denham 1998). No complaints have been received at any of these wind farms. Verve, which operates 8 farms in the state replied “we have never received any form of notification of health complaints in the vicinity of our wind farms.” The three farms in Tasmania have also never received complaints.

Our hypothesis about many wind farms – including those with large turbines – having no history of complaints, with strong spatial (geographical) factors being associated with farms receiving complaints was thus strongly confirmed.

### Hypothesis 2: There would be a Small Proportion of Complaining Residents

Nationally, a total of 129 individuals in Australia appear to have ever formally or publicly complained about wind farm noise or health problems affecting them. Of these, well over half (94 or 73%) came from residents living near just six wind farms (Waubra = 29, McArthur = 21, Hallett 2 = 13, Waterloo = 11, Capital = 10 and Wonthaggi ∼10). Of the remaining farms which have experienced complaints, 9 had between 2 and 6 complainants, and 4 had only single complainants. Of 18 wind farms which had attracted complaints, 11 (72%) have had 6 or less complainants.

There are an estimated 32,789 people living within 5 km of the 50 wind farms for which we obtained residential estimates. Most (20,455 or 62%) live near the 17 smaller wind farms, while 12,334 live within 5 km of the 32 larger farms. In summary, nationally, an estimated 129 individuals have complained out of an estimated 32,789 nearby residents: a rate of about 0.4% or 1 in 254. Of the 34 wind farms with larger (>1 MW) turbines, their 124 complainants represented some 1 in 100 of the surrounding 12,366 residents. Large wind farms with relatively large surrounding rural populations and no histories of complaint include Wattle Point (560), Albany, Starfish Hill (each 200) and Challicum Hills (143).

Again, our hypothesis that the number of complainants living near those wind farms with any history of complaints would be a small proportion of the exposed population, was strongly confirmed.

### Hypothesis 3: Few Wind Farms would have any History of Complaints Consistent with Claims that Turbines cause Acute Effects

Wind farm complainants describe both acute and chronic adverse effects. Acute effects are of particular interest to the psychogenic hypothesis because it is often claimed that even brief exposure to wind turbines can cause almost immediate onset of symptoms. For example, a recent report describes a visit to turbine-exposed houses where people become immediately affected: “The onset of adverse health effects was swift, within twenty minutes, and persisted for some time after leaving the study area” [45]. Symptoms are said to disappear when those affected move away temporarily, only to return as soon as they come back. A highly publicised Lake Bonney complainant who had hosted turbines on his previous property without complaint for six years today claims he and his wife are affected at their new address, further away, but that symptoms disappear as soon as they leave their new home for one or two days [46].

If wind turbine exposure can cause such “instant” problems, any history of delayed or non-reporting of such complaints and the absence of any reports about such complaints in the news media, months or sometimes years after various wind farms began operating creates serious coherency problems for such claims. Such delays would be incompatible with there being widespread or important “acute” effects from exposure.

Table 2 shows that first complaint timing ranged from immediately after turbines commenced operation (sometimes at only a fraction of full capacity) to many months and even many years later (eg: Crookwell, 13.5 years, Lake Bonney, over 7 years later. In five cases (Clements Gap, Hallet 2 & 4, Leonards Hill, Waubra), wind companies advised that complaints anticipating health problems were received before the farms commenced operation. Of the 51 wind farms, 33 (64.7%) have seen no complaints; 6 (11.8%) saw complaints commence at times ranging from 2 months to 13.5 years after turbine operation; and 12 (23.5%) saw either on-going complaints continue from before the wind farms commenced operation or within the first month.

Early complaints from some wind farms could be consistent with acute effects caused directly by turbine exposure but also with nocebo effects caused by anticipation of adverse effects [32]. However, gaps of months or sometimes years between the commencement of turbine operation and complaints are inconsistent with turbines causing acute effects. Moreover, if such effects were serious or common, clinical case reports would have almost certainly appeared in peer reviewed journals, given the many years that wind farms have operated in Australia. No such reports have been published.

### Hypothesis 4: Most Complaints would Date from 2009 or Later, when Anti Wind Farm Groups began to Publicise Alleged Health Effects

The nocebo hypothesis would predict that the spread of negative, often emotive information would be followed by increases in complaints and that without such suggestions being spread, complaints would be less. Australia’s first still operational wind farm commenced operation in 1993 at 10 Mile Lagoon near Esperance, Western Australia. However, objections to wind farms in Australia appear to date from the early years of the 2000 s when press reports mentioned negative reactions of some in rural communities to their intrusiveness in bucolic country landscapes (“behemoths” [47]), bird and bat strikes, the divisiveness engendered in communities by the perceived unfairness of some landowners being paid hosting fees of up to $15,000 per year per turbine while neighbours received none, and debates about the economics of green energy. Unguarded, frank NIMBYism “I’m quite happy to admit that this is a not-in-my-backyard thing, because my backyard is very special” was also evident in 2002 [47].

Groups explicitly opposing wind farms ostensibly because of agendas about preserving pristine bush and rural environments were active from these early years and included many branches of the Australian Landscape Guardians (for example Prom Coast (2002), Spa Country [48], Grampians-GlenThompson [49], Western Plains, Daylesford and District). Key figures in the Landscape Guardians have links with mining and fossil fuel industries [50]. Interests with overt climate change denial agendas also actively opposed wind farm developments, particularly in Victoria. Chief among these were the Australian Environment Foundation, registered in February 2005.

However, health concerns were marginal in these early oppositional years, with one early press report from September 2004 [48] noting “some objectors have done themselves few favours by playing up dubious claims about reflecting sunlight, mental health effects and stress to cattle”.

An unpublished British report said to refer to data gathered in 2003 on symptoms in 36 residents near unnamed English wind farms is frequently noted by global wind turbine opponents as the first known report of health effects from wind turbines, although curiously, it does not appear to have been produced until 2007 [51]. The Daylesford and Districts Landscape Guardians referred to Harry’s work in a 2007 submission opposing a wind farm at Leonards Hill [52].

In Australia, a rural doctor from Toora, Victoria, David Iser, produced another unpublished report [53] in April 2004 following his distribution of 25 questionnaires to households within 2 km of the local 12 turbine, 21 MW wind farm, which had commenced operation in October 2002. Twenty questionnaires were returned, with 12 reporting no health problems. Three reported what Iser classified as “major health problems, including sleep disturbances, stress and dizziness”. Like that of Harry, Iser’s report provides no details of sample selection; whether written or verbal information accompanying the delivery of the questionnaire may have primed respondents to make a connection between the wind turbines and health issues; whether those reporting effects had previous histories of the reported problems; nor whether the self-reported prevalence of these common problems were different to those which would be found in any age-matched population.

In the 10 years between the commencement of operation of the first Esperance wind farm and the end of 2003 when the Harry and Iser health impact reports [51], [53] began being highlighted by turbine opposition groups, 12 more wind farms commenced operation in Australia. In that decade, besides two complainants from Toora, we aware of only one other person living near the north Queensland Windy Hill wind farm who complained of noise and later health soon after operation commenced in 2000. Importantly in that decade, five large turbined wind farms at Albany, Challicum Hills, Codrington, Starfish Hill and Woollnorth Bluff Point commenced operation but never received complaints.

With the exception of those just mentioned and Wonthaggi (∼10 complainants in 2006, but none today) all other health and noise complainants (n = 116) first complained after March 2009– six years after Iser’s Toora small, unpublished survey of health complaints [53] - and particularly from the most recent years when anti wind farm publicity from opposition groups focused on health has grown. Again, the nocebo and the ‘communicated disease’ hypotheses would predict this changed pattern and contagion of complaints, driven by increasing community concern. Sixty nine percent of wind farms began operating prior to 2009 while the majority of complaints (90%) were recorded after this date.

Responding to the nocebo hypothesis and the view that opposition groups were fomenting a ’communicated disease’, the Waubra Foundation’s Sarah Laurie stated: “There is also plenty of evidence that the reporting of symptoms for many residents at wind developments in Victoria such as Toora, Waubra and Cape Bridgewater *preceded the establishment of the Waubra Foundation* (emphasis in original). In the case of Dr David Iser’s patients at Toora the time elapsed is some 6 years.” [54].

This statement neglects to note that the Waubra Foundation’s registration in July 2010 was preceded by several years of virulent wind turbine opposition – which included health claims – by the Landscape Guardians and the Australian Environment Foundation. For example, in November 2009, 8 months before the formation of the Waubra Foundation the Western Plains Landscape Guardians published a full-page advertisement in the local Pyrenees Advocate newspaper headed “Coming to a house, farm or school near you? Wind Turbine Syndrome also known as Waubra Disease”. It listed 12 common symptoms (e.g. sleeping problems, headaches, dizziness, concentration problems). Peter Mitchell is the founding chairman of the Waubra Foundation and in 2009 and at least until February 2011, was also actively advocating for the Landscape Guardians [55].

Table 2 shows that of the 18 wind farms which have seen complainants, 15 (83%) have experienced local opposition from anti wind farm groups. No wind farm with any history of wind turbine opposition avoided at least one health or noise complaint. We conclude that health and noise complaints were rare prior to the decision of anti wind farm groups to focus on these issues and that anti wind farm activists are likely to have played an important role in spreading concern and anxiety in all wind farms areas in which they have been active.

## Discussion

This study shows there are large historical and geographical differences in the distribution of complainants to wind farms in Australia. There are many wind farms, large and small, with no histories of complaints and a small number where the large bulk of complaints have occurred. Just over half of wind farms with larger turbines have seen complaints, but nearly just as many have not. These differences invite explanations that lie beyond the turbines themselves.

Our historical audit of complaints complements recent experimental evidence [32], that is strongly consistent with the view that “wind turbine syndrome” and the seemingly boundless and sometimes bizarre range of symptoms associated with it has important psychogenic nocebo dimensions [2]. While wind turbines have operated in Australia since 1993, including farms with >1 MW turbines from 2001 (Albany and Codrington), health and noise complaints were very rare until after 2009, with the exception of Wonthaggi which saw about 10 complainants in 2006.

Several wind farm operators reported that many former complainants had now desisted. For example, Waubra management advised that not all complainants identified by our public searches had complained to them, and that more than half of the 17 complainant households who had complained to them, had had their complaints resolved. Similarly, Wonthaggi management said that none of some 10 complainants from 2006/2007 were still complaining today. Some of these former complainants from different farms had had their houses noise tested with the results showing they conformed to the relevant noise standard, some received noise mitigation (e.g. double glazing), while others simply stopped complaining.

Opponents sometimes claim that only “susceptible” individuals are adversely affected by wind turbines, using the analogy of motion sickness. Our data produce problems for that explanation: it is implausible that no susceptible people would live around any wind farm in Western Australia or Tasmania, around almost all older farms, nor around nearly half of the more recent farms. No credible hypotheses other than those implicating psycho-social factors have been advanced to explain this variability.

As anti wind farm interest groups began to stress health problems in their advocacy, and to target new wind farm developments, complaints grew. Significantly though, no older farms with non-complaining residents appear to have been targeted by opponents. The dominant opposition model appears to be to foment health anxiety among residents in the planning and construction phases. Health complaints can then appear soon after power generation commences. Residents are encouraged to interpret common health problems like high blood pressure and sleeping difficulties as being caused by turbines.

For example, sleeping problems are very common, with recent Australian and New Zealand estimates ranging from 34% [56], to moderately poor (26.4%) and very poor sleep quality (8.5%) [57]. A German study undertaken to obtain benchmark reference data on common symptoms and illnesses experienced in the past 7 days in the general population for comparison with those experienced by clinical trial enrollees presents data on several problems most often attributed to wind turbines. These include headache (45.3%), insomnia (25.6%), fatigue and loss of energy (19.1%), agitation (18.4%), dizziness (17%) and palpitations (8.6%) [58].

A case brought before The Ontario Environmental Review Tribunal by residents claiming to be affected by a wind farm, collapsed when the Tribunal requested that complaints supply their medical records to determine whether their complaints pre-dated the operation of the wind farm [59].

Wind farm opponents frequently argue complainants are legally “gagged” from speaking publicly about health problems, thus underestimating the true prevalence of those affected. This is said to apply to turbine hosts who are contractually gagged or to non-hosts who have reached compensation settlements with wind companies after claiming harm. The first claim is difficult to reconcile with the example provided by a high profile Lake Bonney wind farm host who continues to complain publicly without attracting any legal consequences [27]. Confidentiality clauses are routinely invoked in any legal settlement to protect parties’ future negotiating positions with future complainants. They usually refer to the settlement figure rather than to the reasons for it.

We purposefully took a liberal view of what a “complainant” was, by including those who had voiced their displeasure about noise, sleep or health in news media or submissions even if they had never lodged a formal complaint with the relevant wind farm company. Despite this, the numbers complaining in Australia were very low and largely concentrated in a small number of “hotbeds” of anti wind farm activism.

A 2012 CSIRO report on nine wind farm developments in three Australian states found widespread acceptance among local residents of both operating and planned farms, and noted that: “The vocal minority are more often prominent in the media … These groups often contact local residents early in the project and share concerns about wind farms.” And that “The reasons for opposition by some participants suggest that wind farms proposals are triggering a range of underlying cultural or ideological concerns which are unlikely to be addressed or resolved for a specific wind farm development. These underlying issues include pre-existing concerns that rural communities are politically neglected by urban centres, commitment to an anti-development stance, and opposition to a ‘green’ or ‘climate action’ political agenda.” [60].

## Limitations

The data we obtained on the number of individuals or occupied houses near the farms were current estimates. These numbers may have varied in different directions for different farms over the 20 year period that wind farms have operated in Australia. But no data are available on that variation. Our estimates of the ratios of complaints to population are therefore unavoidably fixed around the most current population estimates. They would include children who do not lodge complaints, but who are often mentioned by wind farm opponents as subject to health effects [2].

It is possible that there were other complainants who complained earlier than in the periods covered by our corroborative checks. However, this seems highly unlikely: Australian anti wind farm groups would have strong interests in widely publicising such complainants, had they existed. The Waubra Foundation for example, repeatedly refers to the 2004 Iser report [53], in its efforts to emphasise that health concerns had been raised before the Waubra Foundation became established [54] As wind farm opponents have not highlighted more complainants than we have identified, this strongly suggests there were no earlier health or noise complainants.

It is also possible that some of the health complainants are disingenuous, thereby inflating the true number of people actually claiming to experience turbine-related health problems when their objections may be only aesthetic. Controversy arose when an anti wind farm activist who lives 17 km from the Waterloo wind farm was recently accused of “coaching” residents who disliked the local wind farm to explicitly mention health issues [61].

We selected the 5 km distance from turbines as a compromise between the 2 km minimum setback distance designated by the Victorian government for future wind farm approvals, and the 10 km often named by the Waubra Foundation as the advisable minimum distance. We also note here, that one prominent critic of wind farms claims to to be able to personally sense low frequency noise up to 100 km away from wind turbines under certain conditions [62]. Had we chosen the 10 km distance counseled by the Waubra Foundation, this would have significantly increased the numbers of people exposed but not complaining.

The estimates provided by the wind companies of the number of residents within 5 km of wind farms need to be seen as approximations. Census data is available by local government areas and by the Australian Bureau of Statistics statistical regions. However, these do not correspond with the 5 km zone of residence of interest here. The wind companies which provided this data obtained it from their own knowledge of the number of residences near their wind farms and we checked local township sizes from Australian census data. This information is typically obtained during the planning stages of wind farm development when development applications often require such estimations to be provided. At least one company used Google Earth photography to calculate their estimate of the number if dwellings. However, such estimates will always be imprecise and approximations only. They nonetheless provide “ballpark” denominators against which the known number of complainants can be compared.
